# Knowledge and utilization of partograph among obstetric care givers in public health institutions of Addis Ababa, Ethiopia

**DOI:** 10.1186/1471-2393-13-17

**Published:** 2013-01-18

**Authors:** Engida Yisma, Berhanu Dessalegn, Ayalew Astatkie, Nebreed Fesseha

**Affiliations:** 1 Department of Nursing, College of Health Sciences, Samara University, Samara, Ethiopia; 2 Department of Nursing and Midwifery, School of Medicine, College of Health Sciences, Addis Ababa University, Addis Ababa, Ethiopia; 3 School of Public and Environmental Health, College of Medicine and Health Sciences, Hawassa University, Hawassa, Ethiopia; 4 Obstetrician and Gynecologist, National Professional Officer/Making Pregnancy Safer Program WHO Country Office, Addis Ababa, Ethiopia

**Keywords:** Partograph, Knowledge, Utilization, Obstetric care givers, Public health institutions, Addis Ababa, Ethiopia

## Abstract

**Background:**

Globally, there was an estimated number of 287,000 maternal deaths in 2010. Eighty five percent (245,000) of these deaths occurred in Sub-Saharan Africa and Southern Asia. Among the causes of these deaths were obstructed and prolonged labour which could be prevented by cost effective and affordable health interventions like the use of the partograph. The Use of the partograph is a well-known best practice for quality monitoring of labour and subsequent prevention of obstructed and prolonged labour. However, a number of cases of obstructed labour do happen in health facilities due to poor quality of intrapartum care.

**Methods:**

A cross-sectional quantitative study assessed knowledge and utilization of partograph among obstetric care givers in public health institutions of Addis Ababa, Ethiopia using a structured interviewer administered questionnaire. The collected data was analyzed using SPSS version 16.0. Logistic regression analysis was used to identify factors associated with knowledge and use of partograph among obstetric care givers.

**Results:**

Knowledge about the partograph was fair: 189 (96.6%) of all the respondents correctly mentioned at least one component of the partograph, 104 (53.3%) correctly explained the function of alert line and 161 (82.6%) correctly explained the function of action line. The study showed that 112 (57.3%) of the obstetric care givers at public health institutions reportedly utilized partograph to monitor mothers in labour. The utilization of the partograph was significantly higher among obstetric care givers working in health centres (67.9%) compared to those working in hospitals (34.4%) [Adjusted OR = 3.63(95%CI: 1.81, 7.28)].

**Conclusions:**

A significant percentage of obstetric care givers had fair knowledge of the partograph and why it is necessary to use it in the management of labour and over half of obstetric care givers reported use of the partograph to monitor mothers in labour. Pre-service and on-job training of obstetric care givers on the use of the partograph should be given emphasis. Mandatory health facility policy is also recommended to ensure safety of women in labour in public health facilities in Addis Ababa, Ethiopia.

## Background

Globally, there was an estimated number of 287,000 maternal deaths or a maternal mortality ratio (MMR) of 210 maternal deaths per 100,000 live births in the year 2010. Of the estimated total number of 287,000 maternal deaths worldwide, 85% (245,000) occurred in Sub-Saharan Africa and Southern Asia [1]. Maternal mortality ratio continues to be the major index of the widening discrepancy in the level of care and the outcome of reproductive health between the advanced and developing countries [2]. The maternal mortality in Ethiopia is high despite the recognition of maternal mortality as a major public health issue [1]. In Ethiopia, maternal mortality ratio is estimated at 676/100,000 live births [3].

The majority of maternal deaths and complications attributable to obstructed and prolonged labour could be prevented by cost-effective and affordable health interventions like the use of partograph [4,5]. A partograph is one of the valuable appropriate technologies in use for improved monitoring of labour progress, and maternal and foetal wellbeing. It is an important tool for managing labor. This is through enabling midwives, nurses and doctors to record their examination findings on a standardized form, which generates a pictorial overview of labour progress, and maternal and foetal condition, which allows for early identification and diagnosis of pathological labour. Its use is critical in preventing maternal and perinatal morbidity and mortality and therefore has applicability in developed and developing world settings [2].

Most partographs have three distinct sections where observations related to maternal condition, foetal condition and labour progress are recorded [5]. A partograph has clear demarcations which, if arrived at or exceeded clearly indicate the need to address existing or imminent complications like poor progress of labour, prolonged labour, foetal distress, and in the worst cases, obstructed labour and ruptured uterus. Early detection of prolonged labour greatly contributes to prevention of obstructed labor and other related complications such as postpartum hemorrhage (PPH), ruptured uterus, puerperal sepsis and obstetric fistula [6].

The partograph provides health professionals with a pictorial overview of the labour to allow early identification and diagnosis of the pathological labour [7]. The World Health Organization (WHO) recommends using the partograph to follow labour and delivery, with the objectives to improve health care and reduce maternal and foetal morbidity and death [5].

WHO conducted a prospective non-randomized study in South-East Asia and concluded that the partograph was a necessary tool in the management of labour. Findings indicated that introduction and agreed protocol reduced prolonged labour (from 6.4% to 3.4%), the proportion of labour requiring augmentation (from 20.7% to 9.1%), emergency caesarean section (from 9.9% to 8.3%) and still births (from 0.5% to 0.3%) [5,8]. Therefore, proper use of a partograph in an environment where referral and timely intervention are possible would greatly contribute to reduction of maternal mortality and morbidity in the region.

Although the partograph is a simple and inexpensive tool which prevents maternal deaths and complications due to obstructed or prolonged labour, it is not as widely implemented as it should be. Studies from Nigeria did report that only 25% to 33% of caregivers surveyed were using partograph for routine monitoring [2,4]. Caregivers may resist using the tool if they have insufficient knowledge and do not fully understand why they have been asked to use the tool. Non-availability of preprinted partographs has also been reported as a cause for non-utilization [4]. Filling the partograph is also seen as an additional chore for a busy health worker in such a situation and may not be motivated to complete the partograph. However, the challenges to the implementation of the partograph, including insufficient knowledge, non-availability of preprinted partographs and workload pressure, can all be addressed with further education on the purpose of the partograph and local managerial support [4]. Little is known about the knowledge of providers and utilization of partograph; understanding this will be important to inform policies and strategies in provision of maternity care services. Therefore, the objective of this study was to find out obstetric care givers’ knowledge and use of the partograph in the public health institutions of Addis Ababa, Ethiopia.

## Methods

### Study setting

The study was conducted from February 28, 2012 to March 30, 2012 in Addis Ababa, the capital city of Ethiopia and seat of African Union & United Nations World Economic Commission for Africa. Addis Ababa has a population size of over 3 million (3,038,096) with annual growth rate of 2.1% (data obtained from central statistical agency of Ethiopia). The city is divided into ten sub-cities and 100 Kebeles (lowest administrative units in Ethiopia). Addis Ababa is located at 9° 1^′^ 48″ North and 38° 44^′^ 24″ East and the total land area is 54,000 hectares. Its average elevation is 2,500 m above sea level, and hence has a fairly favorable climate and moderate weather conditions.

The city has 48 hospitals. Thirteen are public hospitals of which, 5 are under Addis Ababa Regional Health Bureau (AARHB) and 5 are specialized referral (central) hospitals. Furthermore, the city has 32 health centers under Addis Ababa Health Bureau. There are also two hospitals, three health centers and 31 clinics established by non-government organizations (NGOs), and 33 hospitals and more than 700 clinics that are privately owned.

### Study design

A cross-sectional quantitative study was used to examine knowledge and utilization of partograph among obstetric care givers in public health institutions of Addis Ababa.

### Population

The source population comprised of all health professionals working in maternity units of public health institutions in Addis Ababa. These included midwives, nurses, doctors and public health officers. The study participants were health personnel sampled from the aforementioned categories of health professionals who attend to labour cases in the selected hospitals and health centres and who consented to participate in the study. Health professionals who do not attend labour cases in selected hospitals and health centres were excluded from participating in the study.

### Sampling method

The total number of public health institutions in Addis Ababa is **45** (**13** hospitals and **32** health centres, of which 5 are newly opened health centers). From these institutions, 25 health centres and only 5 public hospitals (i.e., a total of 30 public health institutions) give obstetric care services. The total health personnel who have been providing obstetric care in these 30 institutions are **403** (Data obtained from each institution by the principal investigator via preliminary survey). All institutions which provide obstetric care services were included in the study since the total number of health professionals working in maternity units in the study area was small.

The sample size in this cross-sectional survey was determined using a single proportion formula $n=\frac{Z_{\left(\alpha /z\right)}^{2}p\left(1-p\right)}{w^{2}}$. The minimum sample size required for the study was estimated to be **334** using the above formula where n is the sample size, z is the standard normal deviate set at 1.96 (for 95% confidence level), w is the desired degree of accuracy (taken as 0.05) and p is the estimate of the proportion of our target population who use partograph (assumed to be 32.3% as obtained from study done in the South West Nigeria on Knowledge and utilization of the partograph among obstetric care givers) [2]. The correction formula $n_{f}=\frac{n_{o}}{1+\left(\frac{n_{o}}{N}\right)}\text{,}$ where n_*f*_ is the final sample size, n_o_ is the initial sample size and N is the total number of obstetric care givers in public health institutions of Addis Ababa, was used as the total number of health professionals working in the maternity units of public health institutions of the study area was less than 10,000. Consequently, the corrected sample size became 183. Adjustment for a 10% rate of non-responses yielded a final sample size of **202**.

The sample was allocated to the institutions included in the study proportional to the number of health professionals working in the maternity units of the respective health institutions. And at each study center simple random sampling was employed to select the eligible health professional for the interview.

### Data collection

#### Instrument

A pre-tested and structured, interviewer administered questionnaire was adapted from previous study [2] and reviewing relevant literature to the problem under study to include all the possible variables that address the objective of the study. The questionnaire contained a combination of open ended and closed ended questions. The questionnaire was designed to obtain information on the professional characteristics of the obstetric care givers, awareness of the partograph, main source of knowledge of the partograph, its benefit and whether or not they routinely employ it in labour management. There were also questions that assessed the depth of their knowledge of the partograph and their interest in being trained in its use. In order to produce a more objective assessment of knowledge of the partograph, a scoring method was devised and a ‘knowledge score’ for each of the personnel was obtained by adding up the scores for correct answers given to selected questions in the questionnaire [9]. The criteria for scoring knowledge are displayed in Table 1.

**Table 1:** Criteria for the partograph knowledge score

**Parameters**	**No**	**Yes**
Awareness of partograph	0	2
Correct definition of the partograph	0	3
Knows the benefit of the partograph to parturient	0	2
**Knowledge of observations on the partograph**		
Cervical dilatation	0	3
Foetal heart rate	0	2
Uterine contraction	0	2
Descent of the presenting part	0	2
Maternal blood pressure	0	2
Maternal pulse	0	2
Color of liquor	0	2
Maternal temperature	0	2
Oxytocin regimen	0	2
Intravenous fluids & drugs	0	2
Urine test results	0	2

align="center"Minimum score: 0; Maximum score: 30. Scores, 0 – 10, poor level of knowledge; 11 – 20, fair level of knowledge; 21 – 30: good level of knowledge [9].

Based on the overall knowledge scores, the respondents’ level of knowledge of the partograph was rated as poor (0 – 10), fair (11 – 20) and good (21 – 30). These knowledge ratings were based on obtainable scores by the respondents, which we believed portrayed increasing understanding of the partograph. A score of at least 10 was obtainable by having a basic knowledge of the partograph: awareness of the partograph (**2**), knowledge of its correct definition (**3**), its benefits (**2**), and cervical dilatation as an observation on the partograph (**3**). A score of 11 – 20 will be obtainable if the respondents had this basic knowledge in addition to knowing the important observations on the partograph such as foetal heart rate (**2**), uterine contractions (**2**), descent of the presenting part (**2**), maternal pulse (**2**) and maternal blood pressure (**2**). A score of more than 20 will be obtainable if the respondents knew almost/all of the observations recorded on the partograph in addition to having its basic knowledge [9].

#### Data analysis

Data entry and analysis were performed using Epi Info Version 3.5.1 software and SPSS version 16. The data entered were checked for their consistency. Data cleaning was done using find and sort commands of Epi info. Frequency distributions and cross tabulations were used to describe the variables of the study. The relationship between selected independent variables and the respondents’ utilization of partograph and level of knowledge were explored using bivariate and multivariate logistic regression analysis. The presence and magnitude of association was checked using the odds ratio (OR) with 95% CI. Observed differences between samples were considered statistically significant where the confidence limits did not embrace unity or p < 0.05.

#### Ethical considerations

Permission was obtained from the Ethical Clearance Committee of Addis Ababa University, College of Health Sciences, School of Medicine, Department of Nursing and Midwifery. Protection of the rights of the study participants was ensured by giving them due freedom to participate in the study or not to participate. Privacy and confidentiality were maintained during interview. The subjects were told any information they provided would be kept confidential. Their names never appeared on data collection tools. Additionally, the study subjects were informed that their responses would not bring any harm to them and wouldn’t in any way affect their job.

At each of the selected study sites, the matron/medical director was contacted for permission and necessary information before the commencement of the study. The purpose, general content and nature of the investigation were explained to each respondent to obtain a verbal and written consent before inclusion into the study.

## Results

### Characteristics of the interviewed obstetric care givers

Out of the 202 questionnaires that were administered, 195 questionnaires were correctly completed making a response rate of 96.5%. The respondents comprised of 88 (45.1%) midwives, 76 (39.0%) nurses, 19 (9.7%) physicians and 12 (6.2%) public health officers. Sixty one (31.3%) of the respondents were from health centres and 134 (68.7%) were from hospitals. Most of the respondents were midwives while the least respondents, public health officers, constituted one-sixteenth of the respondents. The mean and standard deviation of the age of the respondents were 29.14 and 6.93 years respectively. The median age was 27 years (range: 21–57 years). The duration of practice ranged between 1 and 34 years with the mean (±standard deviation) and median duration of 7.06 (±6.52) and 5 years respectively. A total of 115 (59.0%) of the participants had been practicing for < 5 years, while 43 (22.1%), 21 (10.8%) and 4 (2.1%) of them had been in practice for 6–10, 11–15 and 16–20 years respectively. Some 12 participants (6.2%) had been in practice for over 21 years (See Table 2).

**Table 2:** **Characteristics of the interviewed obstetric care givers**, **public health institutions of Addis Ababa**, **2012**

**Variable**	**Hospital**		**Health centre**		**Total**	
	**n**	**%**	**n**	**%**	**n**	**%**
**Professional qualification** (n = 195)						
Midwife	35	39.6	53	60.2	88	100.0
Nurse	5	6.6	71	93.4	76	100.0
Public Health Officer	2	16.7	10	83.3	12	100.0
Medical Doctor	19	100.0	0	0.0	19	100.0
**Sex** (n = 195)						
Male	21	34.4	40	65.6	61	100.0
Female	40	29.9	94	70.1	134	100.0
**Level of Education**						
Diploma	17	16.7	85	83.3	102	100.0
Bachelor of Science	25	33.8	49	66.2	74	100.0
Medical Doctor	8	100.0	0	0.0	8	100.0
Medical Doctor plus Specialization	11	100.0	0	0.0	11	100.0
**Professional tenure**						
Less than 5 years	45	39.1	70	60.9	115	100.0
5-10 years	9	20.9	34	79.1	43	100.0
11-15 years	4	19.0	17	81.0	21	100.0
16-20 years	1	25.0	3	75.0	4	100.0
21 or more years	2	16.7	10	83.3	12	100.0
**Total**	61	31.3	134	68.7	195	100.0

### Awareness and knowledge of the partograph among obstetric care givers

All of the 195 (100%) respondents knew what a partograph was. Of those who knew what a partograph was, 119 (61.0%) respondents knew its correct definition, 56(28.7%) respondents reported incorrect definition of the partograph and 20 (10.3%) respondents didn’t know the definition of the partograph at all. Knowledge of the function of both alert line and action line were poor. Only 104 (53.3%) respondents could correctly explain the function of alert line while 53 (27.2%) and 38 (19.5%) of the respondents gave incorrect explanation and didn’t know the correct function of alert line at all respectively. One hundred and sixty one (82.6%) of the respondents could correctly explain the function of action line while 16 (8.5%) of the respondents couldn’t correctly explain the function of action line and 18 (9.2%) of the respondents didn’t know the function of action line at all.

One hundred eighty nine (96.6%) of the respondents could correctly mention at least one component of the partograph. There was no statistically significant difference between respondents from hospitals and health centres regarding correctly mentioning at least one component of the partograph [Crude OR = 2.3 (95% CI: 0.44, 11.50)]. More respondents (54 (88.5%)) from hospitals could explain the function of action line compared with 107 (79.9%) respondents from health centres but more respondents (131 (97.8%)) from health centres could correctly mention at least one component of the partograph compared with 69 (51.5%) respondents from hospitals.

Knowledge of obstetric care givers about assessment of labour with the partograph was also investigated (Table 3). Most (191 (97.9%)) of respondents reported that they can make diagnosis of prolonged labour using partograph while only 123 (63.1%) of the respondents knew about dehydration in mother as an assessment that could be inferred from the partograph during labour.

**Table 3:** **The proportion of obstetric care givers who correctly identified the components of labour assessment**, **public health institutions of Addis Ababa**, **2012**

**Knowledge of assessment of Labour with partograph**	**Frequency**	**%**
	**(n = 195)**	
Prolonged labour	191	97.9
Obstructed labour	149	76.4
Poor progress of labour	186	95.4
Inefficient uterine contraction	173	88.7
Suspected foetal distress	173	88.7
Abnormal foetal heart rate	179	91.8
Satisfactory progress of labour	176	90.3
Need for augmentation of labour	170	87.2
Need for caesarean section	148	75.9
Dehydration in mother	123	63.1

About 117 (60.0%) of the respondents agreed that in normal progress of labour, plot on the partograph falls on the left of alert line, 49 (25.1%) disagreed while 29 (14.9%) did not know. The result further indicate that out of the 195 respondents, 129 (66.2%) agreed that it would fall on the alert line, 35 (17.9%) disagreed while 31 (15.9%) did not know. Moreover, 67 (34.4%) of the respondents agreed that in the normal progress of labour, graph/plot on the partograph would fall on the right of alert line, 96 (49.2%) disagreed while 32 (16.4%) did not know.

More than half (102 (52.3%)) of respondents had fair knowledge of partograph, while less than half (79 (39.0%)) of them had good knowledge of it and 17 (8.7%) had poor knowledge of partograph. Only one-fourth of the public health officers had good level of knowledge while slightly more than half (53.3%) of the midwives were rated as having good level of knowledge and most (68.4%) of the nurses were rated as having fair level of knowledge.

University/college was reported as a primary source of knowledge by the majority (92 (53.2%)) of those who were aware of the partograph and almost all of these individuals had fair and good level of knowledge. Ninety (80.4%) of those who were aware of the partograph employed it routinely in labour management.

### Bivariate and multivariate logistic regression analysis of factors associated with knowledge of obstetric care givers about partograph

Table 4 below shows the association of some selected professional characteristics and other variables with knowledge of obstetric care givers about partograph. On crude logistic regression analysis, a significantly higher proportion of obstetric care givers working in hospitals had a good level of knowledge about the partograph compared to those working in health centres [Crude OR = 2.0 (95% CI: 1.1, 3.9)]. However, on multivariate logistic regression analysis this association was not significant [Adjusted OR = 1.05 (95% CI: 0.33, 1.69)].

**Table 4:** Bivariate and multivariate logistic regression analysis of knowledge on partograph

**Characteristics**		**Overall knowledge**		
	**Poor**	**Good**	**Crude OR(95% CI)**	**Adjusted OR(95% CI)**
	**n(%)**	**n(%)**		
Sex				
**Male**	34(55.7)	27(44.3)	1.4(0.7,2.5)	1.05(0.49, 2.23)
**Female**	85(63.4)	49(36.6)	1	1
Types of institution				
**Hospital**	30(49.2)	31(50.8)	**2**.**0**(**1**.**1**,**3**.**9**) *	0.74 (0.33, 1.69)
**Health centre**	89(66.4)	45(33.6)	1	1
Profession				
**Midwife**	41(46.6)	47(53.4)	0.5(0.2,1.5)	0.41(0.11,1.47)
**Nurse**	63(82.9)	13(17.1)	**0**.**1**(**0**.**03**,**0**.**97**) *	**0**.**07**(**0**.**02**, **0**.**31**) *
**Public health officer**	9(75.0)	3(25.0)	**0**.**2**(**0**.**03**,**0**.**78**) *	0.10(0.02, 0.67)
**Doctor**	6(31.6)	13(68.4)	1	1
Years of service				
**5 years and less**	70(60.9)	45(39.1)	1.6(0.6,1.8)	0.87(0.44,1.74)
**Over 5 years**	49(61.2)	31(38.8)	1	1
Previous training				
**Yes**	12(37.5)	20(62.5)	**3**.**2**(**1**.**45**,**6**.**98**) *	**2**.**82**(**1**.**19**, **6**.**70**) *
**No**	107(65.6)	56(34.4)	1	1

Note: * statistically significant at 95% CI, P < 0.05; 1 = reference.

Similarly, on crude analysis nurses compared to medical doctors had a lesser likelihood of having a good level of knowledge about the partograph [Crude OR = 0.1 (95% CI: 0.03, 0.97)]. This association remained significant after adjustment for possible confounding variables using multivariate logistic regression [Adjusted OR = 0.07 (95% CI: 0.02, 0.31)].

Previous training about the partograph also showed significant association with level of knowledge about the partograph. More of those who had previous training on partograph had a good level of knowledge compared to those who never had formal training (Adjusted OR = 2.8 (95% CI: 1.19, 6.70)).

Other variables such as sex and professional tenure of the obstetric care givers and perception of the obstetric care givers about the partograph didn’t show any statistically significant association with the level of knowledge of the obstetric care givers about the partograph (See Table 4).

### Utilization of partograph among obstetric care givers in public health institutions

Only 112 (57.4%) of the respondents used the modified WHO partograph to monitor women in labour in public health institutions of Addis Ababa, Ethiopia. The use of the partograph was reported significantly more frequently by respondents in the health centres compared with the respondents from hospitals (67.9% vs. 34.4%; (χ ^2^ = 19.2, df =1, p < 0.01). Among those who used partograph, 90 (80.4%) used it routinely while 10 (8.9%) and 12 (10.7%) of respondents used it occasionally and sometimes respectively (See Table 5).

**Table 5:** The practice of partograph among obstetric care givers

**Variables**	**Frequency**	**%**
**Do you use partograph** (n = 195)		
Yes	112	57.4
No	83	42.6
**How often do you use**? (n = 112)		
Routinely	90	80.4
Sometimes	10	8.9
Occasionally	12	10.7
**Total**	112	100.0

### Reasons for not using the partograph

Among participants who were aware of the partograph but never used it in monitoring of labour, reasons for not routinely using it were cited as little or no knowledge of the partograph (30.66%), much detail to fill (10.48%), time consuming (28.23%), lack of adequate number of personnel (6%), ‘doctors do that’ (16.13%), and lack of training (8.06%). A total of 155 (95.1%) of those who were aware of the partograph desired training on its use while 8 (4.9%) of them did not have any desire for training.

### Bivariate and multivariate logistic regression analysis of factors associated with partograph utilization

Obstetric care givers working in health centres had significantly higher odds of utilizing the partograph compared to those working in hospitals [Adjusted OR = 3.63 (95% CI: (1.81, 7.28)] while more of those obstetric care givers who had not been previously trained on the partograph had lesser odds of utilizing the partograph compared to those who had been previously trained [Adjusted OR = 0.39 (95% CI: 0.16, 0.97)].

Likewise, obstetric care givers who lacked positive attitude about the partograph had lesser odds of utilizing the partograph in monitoring of mothers in labour compared to those who had positive attitude towards the partograph [Adjusted OR = 0.10 (95% CI: (0.01,0.81)].

Similar to the crude analysis, the multivariate analysis of obstetric care givers’ sex, i.e., being female [Adjusted OR = 0.91 (95% CI: (0.46, 1.80)] compared to being male and those obstetric care givers with experience of more than 5 years [Adjusted OR = 1.02 (95% CI: (0.53, 1.94)] compared to those with experience of 5 years or less showed no significant associations (See Table 6).

**Table 6:** Bivariate and multivariate logistic regression analysis of factors associated with partograph utilization

**Characteristics**		**Overall utilization**		
	**Utilized**	**Not utilized**	**Crude**	**Adjusted**
	**n(%)**	**n(%)**	**OR(95% CI)**	**OR(95% CI)**
Sex				
**Male**	35(57.4)	26(42.6)	1	1
**Female**	77(57.5)	57(42.5)	1.00(0.54,1.85)	0.91(0.46,1.80)
Types of institution				
**Hospital**	21(34.4)	40(65.6)	1	1
**Health centre**	91(67.9)	43(32.1)	**4**.**03**(**2**.**12**,**7**.**65**)*	**3**.**63**(**1**.**81**,**7**.**28**)*
Years of service				
**5 years or less**	62(53.9)	53(46.1)	1	1
**Over 5 years**	50(62.5)	30(37.5)	1.43(0.80,2.55)	1.02(0.53,1.94)
Previous training				
**Yes**	24(75.0)	8(25.0)	1	1
**No**	88(54.0)	75(46.0)	**0**.**39**(**0**.**17**,**0**.**92** *	**0**.**39**(**0**.**16**,**0**.**97**)*
Do you like partograph?				
**Yes**	111(61.0)	71(39.0)	1	1
**No**	1(7.7)	12(92.3)	**0**.**05**(**0**.**01**,**0**.**42**)*	**0**.**10**(**0**.**01**,**0**.**81**)*

Note: * statistically significant at 95% CI, P < 0.05; 1 = reference.

## Discussions

This study focused on obstetric care givers in public health institutions of Addis Ababa, Ethiopia, specifically, at delivery units. The study participants were selected from hospitals and health centres. Findings from the survey may therefore be regarded as a window that provides a glimpse into current knowledge base and the obstetric care practice within the study area.

In this study, all of the 195 (100%) respondents knew what a partograph was and more than half of the respondents 102 (52.3%) had fair knowledge of the partograph while only 79 (39.0%) of them had good knowledge of it. This result implies that knowledge of obstetric care givers on partograph may be inadequate for better utilization of partograph in public health institutions and this seems somewhat comparable with the same study done in Nigeria [9] in which more than half of the respondents had fair knowledge of the partograph, while less than one-third of them had a good knowledge of it. Moreover university/college was reported as the primary source of knowledge by the majority (53.2%) of those who were aware of the partograph in this study but life-saving skill training workshop was reported as the primary source of knowledge by one-third of those who aware of the partograph in a study done in Nigeria [9]. This might justify the reason why the majority (83.6%) of the respondents in public health institutions of Addis Ababa, Ethiopia had not received training.

Knowledge of the function of alert line compared with action line was poor. Only 104 (53.3%) of the respondents could explain the function of alert line while 161(82.6%) of the respondents could explain the function of action line, which may indicate the need for very urgent steps to improve the knowledge of obstetric care givers on the partograph through training and seminars in order to maximize the utilization and proper use of the partograph. This result, however, shows higher figure compared with a study done in Nigeria [2] in which about 119 (16.6%) of the respondents could explain the function of alert line while 175 (24.3%) could explain the function of action line. This could be due to the fact that most respondents in the present study were midwives. It could also be due to differences in the approaches of training obstetric care givers in Ethiopia and Nigeria.

Though most (52.3%) respondents in this study had fair knowledge of the partograph, there was poor utilization of it in labour monitoring considering the WHO recommendation for partograph use in public health institutions [5]. Several similar studies confirmed the low utilization of the partograph in Africa [2,10-13]. Inadequate knowledge and utilization of partograph could be part of the reasons for high maternal mortality in Ethiopia and other developing countries [1,3]. This necessitates the need for regular pre-service and on-job training of obstetric care givers on use of the partograph for safety of women in labour.

In this study, the utilization of the partograph was significantly higher among obstetric care givers working in health centres (67.9%) compared to those working in hospitals (34.4%) [Adjusted OR = 3.63 (95% CI: 1.81, 7.28)]. This is in line with the study done in Ogun state, Nigeria [9] where the tool was not uniformly utilized in the settings where it was most needed (at secondary health hospitals and private health institutions) [9,10]. This may be due to the fact that at health centres more obstetric care givers use the partograph regularly to identify abnormal labour patterns early and arrange for the timely referral to higher centres though what is required is that all health facilities, whether hospitals or health centres, should regularly use the partograph to monitor mothers in labour.

There was no significant relationship between the years of service of the study participants as obstetric care givers and their use of the partograph [Adjusted OR = 1.02 (95% CI: 0.53, 1.94)]. This finding points to the need that obstetric care givers should get periodic on-job refresher trainings on the use of partograph. In such endeavours, obstetric care givers with longer years of service should not be overlooked assuming that their accumulated experience would enable them to better make use of the partograph.

This study also revealed that only 32 (16.4%) of the respondents received in-service training on the partograph of whom more than half utilized the partograph in monitoring of women in labour. This finding is contrary to the study done in Dar es Salaam, Tanzania [12] where all midwives interviewed had been previously trained in-service to use the partograph. Though all respondents in our study mentioned that they had formal education on the partograph while they were in colleges/universities, about 95% of them demanded in-service training to be able to use the partograph effectively which implies that the pre-service training alone is not enough to effectively make use of the partograph in practice.

Additionally, almost all (97.9%) of the respondents in our study admitted that the use of the partograph could prevent prolonged labour and facilitate early referral to specialized health facilities. This goes in line with the findings that showed the partograph to be an efficacious tool for monitoring labour and identifying women in need of an obstetric intervention [8,12]. WHO had also showed in its safe motherhood programme that the partograph is effective in reducing prolonged labour, in reducing rate of labour augmentation, in reducing rate of caesarean sections and also in reducing the number of still births [5].

The limitations of this study could include the following. Firstly, there might be social desirability bias which may cause the obstetric care givers who took part in this study to overstate their use of the partograph. Secondly, as this study is confined to obstetric care givers working in public health facilities of Addis Ababa, Ethiopia, the findings may not be generalizable to obstetric care givers working in private health facilities as well as in public and private health facilities out of Addis Ababa. The other limitation of the study could be the small sample size which may make estimates unstable and associations between dependent and independent variables undetectable.

## Conclusions

More than half (53.3%) of obstetric care givers in public health institutions of Addis Ababa have fair knowledge of partograph and why it is necessary to use it in the management of labour. A significant percentage of the respondents thought that using the partograph would improve the maternal and neonatal morbidity and mortality situation in the country. Over half of the obstetric care givers reported that they used the partograph to monitor mothers in labour. Use of the partograph during labour was affected by factors like lack of knowledge, lack of training of obstetric care givers on the use of the partograph and obstetric care givers’ lack of positive attitude towards the use of the partograph. Working in the health centres was significantly related to the utilization of the partograph.

Based on the findings of this study, pre-service and periodic on-job training of obstetric care givers on the use of the partograph, regular supportive supervision and mandatory health facility policy are recommended for the safety of mothers in labour in Addis Ababa, Ethiopia.

## Abbreviations

AARHB: Addis Ababa Regional Health Bureau; MMR: Maternal Mortality Rate; NGOs: Non-governmental Organizations; PPH: Post-Partum Hemorrhage; OR: Odds Ratio; WHO: World Health Organizations.

## Competing interests

The authors declare that they have no competing interests.

## Authors’ contributions

All authors (EY, BD, AA, and NF) contributed to the design of the study and the interpretation of data. EY performed the data analysis and drafted the manuscript. All other authors (BD, AA, NF) critically revised the manuscript and have approved the final version. All authors read and approved the final manuscript.

## Pre-publication history

The pre-publication history for this paper can be accessed here:

http://www.biomedcentral.com/1471-2393/13/17/prepub
